# Alteration of the Cortex Shape as a Proxy of White Matter Swelling in Severe Cerebral Small Vessel Disease

**DOI:** 10.3389/fneur.2019.00753

**Published:** 2019-07-10

**Authors:** François De Guio, David Germanaud, Julien Lefèvre, Clara Fischer, Jean-François Mangin, Hugues Chabriat, Eric Jouvent

**Affiliations:** ^1^Université Paris Diderot, UMR-S 1161 INSERM, Paris, France; ^2^DHU NeuroVasc Sorbonne Paris Cité, Paris, France; ^3^Université de Paris, Inserm, NeuroDiderot, inDev Team, Paris, France; ^4^CEA, NeuroSpin, UNIACT, Gif-sur-Yvette, France; ^5^AP-HP, Hôpital Robert-Debré, Service de Neurologie Pédiatrique et des Maladies métaboliques, Paris, France; ^6^Institut de Neurosciences de la Timone, CNRS UMR7289, Aix-Marseille University, Marseille, France; ^7^UNATI, NeuroSpin, I2BM/DSV, CEA, Paris Saclay University, Paris, France; ^8^AP-HP, Lariboisière Hospital, Department of Neurology, Paris, France

**Keywords:** cortex, shape, white matter hyperintensites, small vessel disease, CADASIL

## Abstract

CADASIL is a monogenic small vessel disease characterized by the accumulation of brain tissue lesions of microvascular origin leading to strokes and cognitive deficits. Both cortical and parenchymal alterations have been described using various MRI markers. However, relationships between cortical and subcortical alterations remain largely unexplored. While brain atrophy is a preponderant feature in cerebral small vessel disease, recent results in CADASIL suggest slightly larger brain volumes and increased white matter water content at early stages of the disease by comparison to controls. We hypothesized in this study that increased water content in gyral white matter balances expected brain atrophy. Direct white matter volume computation is challenging in these patients given widespread subcortical alterations. Instead, our approach was that a gyral white matter swelling would translate into a modification of the shape of cortical gyri. Our goal was then to assess the relationship between subcortical lesions and possible alteration of the cortex shape. More specifically, aims of this work were to assess 1) morphometric differences of the cortex shape between CADASIL patients and controls 2) the relationship between the cortex shape and the volume of white matter hyperintensities (WMH), a reflect of white matter alterations. Twenty-one patients at the early stage of the disease and 28 age- and sex-matched controls were included. Cortical surfaces were reconstructed from 3D-T1-weighted images. Folding power assessed from spectral analysis of gyrification and cortical morphometry using curvedness and shape index were computed as proxies of the cortex shape. Influence of segmentation errors were evaluated through the simulation of WMH in controls. As a result, patients had larger folding power and curvedness compared to controls. They also presented lower shape indices both related to sulci and gyri. In patients, the volume of WMH was associated with decreased gyral shape index. These results suggest that the cortex shape of CADASIL patients is different compared to controls and that the enlargement of gyri is related to the extent of white matter alterations. The study of the cortex shape might be another way to evaluate subcortical swelling or atrophy in various neurological disorders.

## Introduction

CADASIL (Cerebral Autosomal Dominant Arteriopathy with Subcortical Infarcts and Leukoencephalopathy) is a monogenic brain disorder, due to *NOTCH3* mutations, characterized after a normal brain development, by the accumulation of brain tissue lesions of microvascular origin as soon as the 3rd or 4th decade (1). While the accumulation of tissue lesions is usually expected to be associated with the development of brain atrophy in cerebral small vessel disease, we recently observed in patients at early stage of the disease that brain volume did not differ from that of age- and sex-matched healthy individuals (2). We also found with the use of T1 and T2^*^ relaxometry that the white matter water content was presumably increased in patients, particularly in gyral juxtacortical areas (3), which led us to hypothesize that brain volume loss might be absent or masked by an increase of white matter water content (swelling) at the initial stage of the disorder.

**Figure 1:**
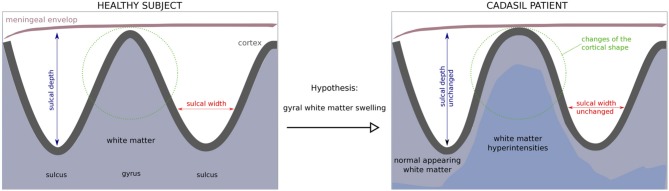
Hypothesis of a gyral white matter swelling in CADASIL and its consequences on the cortex shape. A gyral white matter swelling in CADASIL would be congruent with a modification of the curvedness and the shape index that quantify 3D-cortex morphology. Conversely, estimates such as cortical thickness, sulcal depth/width or gyrification index are not suitable to detect such changes in the gyral shape.

Given that tissue lesions accumulate mainly in deep brain areas, while the increase of white matter water content seems to predominate in certain juxtacortical areas, we aimed to determine whether the gyral white matter was actually increased when compared to age- and sex-matched controls. Unfortunately, the gray to white matter tissue contrast may be altered in CADASIL (4) and the segmentation of gyral white matter is hampered by the presence of extensive white matter lesions and the difficulty to distinguish gyral vs. sulcal regions. Hence, a reliable comparison of white matter volume with that of controls is difficult. Another pathway to test the hypothesis of a gyral white matter swelling is to consider the shape of the cortex as a proxy of its underlying white matter, given that the gyrification pattern is sensitive to geometrical and mechanical perturbations (5). In the present study, we hypothesized that a gyral white matter swelling would translate into a modification of the shape of cortical gyri. As schematized in Figure 1, the white matter beneath the gyri was supposed to be enlarged, making the cortical gyri more sharpened and wide as constrained by the meningeal envelope and the skull (6).

**Figure 2:**
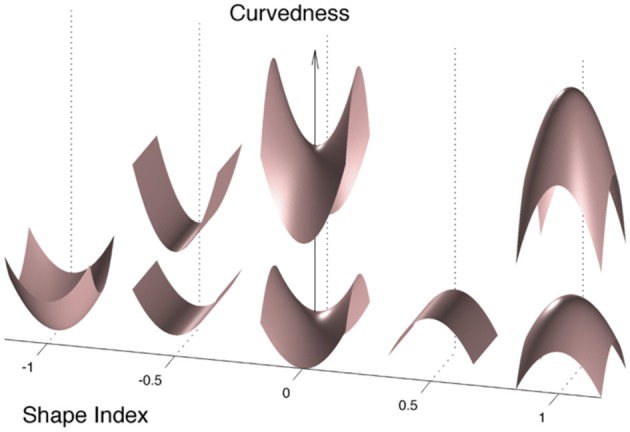
Illustration of shape index and curvedness. The Curvedness represents the power of folding and the Shape Index describes the local aspect of the surface. The Shape Index is bounded between −1 and 1, from a sulcal pit (−1) or a local bump (1) to an archetypal sulcus (−0.5) or an archetypal gyrus (0.5).

While these fine shape alterations are out of reach of advanced computational tools such as cortical thickness estimation, gyrification indices or sulcal morphometry previously used in CADASIL (7, 8) (Figure 1), it can be captured by using local curvature-based descriptors such as the curvedness and the shape index that have recently emerged and bring additional information on the 3-D cortical morphology (Figure 2). For example, as illustrated in Figure 1, sulcal depth or sulcal width are thought to be insensitive measures of a gyral white matter swelling by contrast to the curvedness and shape index which are based on the local curvature of the cortical surface. More specifically, both descriptors derived from surrogates of the two local principal curvatures, the curvedness representing the local bending intensity (sharpness), while the shape index describing the local aspect of the surface (from pit to saddle shape). Computation of those metrics has been notably used to quantify morphological changes in the developing brain (9–11). Another new approach is to apply a Fourier-like analysis to the variations of the curvature on a cortical mesh (12). This spectral analysis of gyrification (SPANGY) provides a frequency analysis of the folding pattern that has been used to quantify morphological changes coming with size variations among healthy adults and between microcephalic patients (13).

Finally, to ensure that these potential cortical alterations are actually secondary to the disease evolution and not due to innate differences with controls, we tested the relationships between cortical metrics and white matter hyperintensities (WMH), a well admitted reflect of white matter alterations in this disorder.

The aims of this work were then to (1) detect subtle morphometric differences between CADASIL patients and age- and sex-matched controls using geometrical and spectral descriptors of the cortex shape (2) if present, to infer the relationship between cortex shape changes in patients and the volume of WMH.

We included in our analysis other factors that may impact the cortex shape to better highlight the independent effect of WMH. Thus, volume of lacunes were computed given their potential role in cortical thinning and on macroscopic cortical abnormalities through secondary cortical neurodegeneration (14, 15). Also, we paid a particular attention to brain tissue segmentation errors that could bias the measurement of curvature-based descriptors. To that purpose, a simulation was performed to generate WMH in cerebral images obtained in the control population as these lesions were supposed to interfere with shape measurements.

## Materials and Methods

### Participants

Non-demented non-disabled (MMSE score ≥24 and modified Rankin's scale ≤1) adult CADASIL patients at the early clinical stage of the disease were recruited from the CADASIL cohort followed in the National Referral Center for this disorder. Controls were drawn from a local database of healthy volunteers free of any known history of neurological disorder and without symptoms of cognitive impairment or disability as evaluated by a structured interview. Twenty-one patients and 28 controls having good image and cortical surface reconstruction qualities were included in this study.

This study was carried out in accordance with the recommendations of the ethics committee of DRCD (Département de la Recherche Clinique et du Développement) of AP-HP (Assistance Publique-Hôpitaux de Paris) with written informed consent from all subjects. All subjects gave written informed consent in accordance with the Declaration of Helsinki. The protocol was approved by the DRCD of AP-HP.

### Magnetic Resonance Imaging Protocol

3D T_1_-weighted images used for cortical surface reconstruction were obtained at 3 Tesla with a Tim-Trio MRI scanner (Siemens Healthcare, Erlangen, Germany) equipped with a 12-channel head coil, using a standard sagittal magnetization-prepared rapid acquisition gradient echo (MPRAGE) sequence (in plane resolution: 1 × 1 mm^2^, slice thickness = 1.1 mm, TR = 2,300 ms, TE = 2.98 ms, TI = 900 ms, FA = 9°, BW = 238 Hz/pixel, time of acquisition = 7′45 min). Volumes of lacunes and of WMH of presumed vascular origin were determined from 3D T1-weighted and FLAIR images obtained within 6 months on a 1.5T Signa scanner (GE Healthcare, Milwaukee, Wisconsin).

### Image Processing

The analysis was restricted to the left hemisphere. All subjects were right-handed.

#### Subcortical Lesion Quantification

Delineation of WMH and of lacunes was performed according to the STRIVE criteria (16). Masks of WMH and of lacunes were obtained for each patient from FLAIR and 3DT1 sequences, respectively. The volume of WMH and of lacunes was determined by multiplying the number of voxels in WMH mask or in lacunes mask by voxel size as previously reported (7).

#### Cortical Surface Reconstruction and Global Morphometry

The Morphologist pipeline of BrainVISA (http://www.brainvisa.info) was used to automatically segment the left cortical surfaces (mesh of the gray-white interface) from 3D T1-weighted images (17). In patients, prior to segmentation, masks of WMH were registered to 3D T1-weighted images and, as previously reported (8), voxel intensity inside WMH was set up to an average intensity close to that of normal-appearing white matter to overcome segmentation difficulties possibly induced by WMH in patients. For each subject, BrainVISA was also used to compute the left brain volume *BV* defined as the sum of gray matter and white matter volumes, the left surface area *SA*, the hemispheric hull area *HA* that is the morphological closing of the hemispheric mask, and the total volume inside the hull *HV*. The global gyrification index (*GGI*) was defined as the ratio of surface area to hull area (GGI = SA / HA) and the spherical gyrification index (*SGI*) was calculated as the ratio of surface area to the area of a sphere of volume equals to the brain volume (SGI = SA/4π ^*^(3BV/4π)2/3) (18). The normalized brain volume was computed as the ratio of both hemispheres *BV* to *HV*.

#### Curvature-Based Cortex Shape Analysis

**Figure 3:**
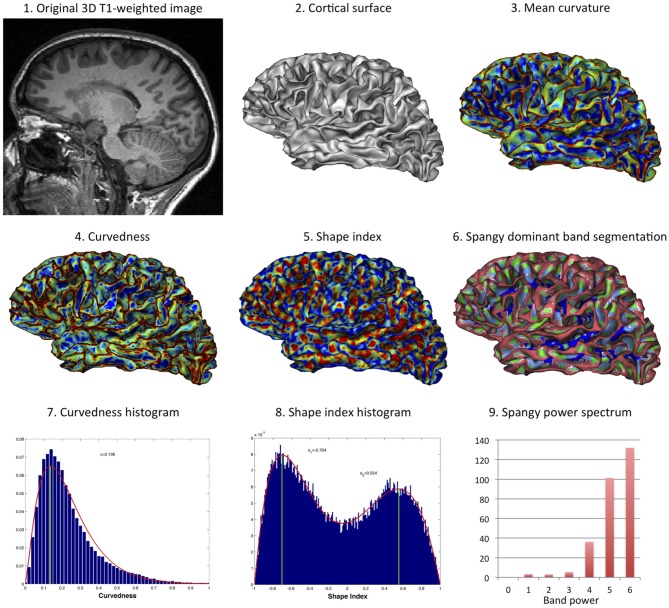
Computational methods for curvature-based cortex shape analysis. From an original 3D T1-weighted image (1), the cortical surface was extracted using BrainVisa (2). The mean curvature has been computed vertex-wise (3), so as the curvedness (4), and the shape index (5). The spectral analysis of gyrification can be represented through the dominant band segmentation (the power band that essentially determines the local curvature) (6). For simple analysis at the whole-brain scale, histograms of the curvedness (7) and of the shape index (8) have been modeled by a gamma distribution and a mixture of two beta distributions, respectively. The band power of each major band of the spectrum (B4, B5, B6) has been further used in the analysis (9).

Figure 3 illustrates the various computational methods used for cortex shape analysis.

The spectral analysis of gyrification was performed using the Spangy method (12). Briefly, the method consists in the spectral analysis of the mean curvature spatial variations taken as a proxy of the cortical folding pattern. It achieves a quantitative analysis of cortical folding pattern both in the frequency domain thanks to a band power spectrum and in the image domain where each piece of sulcus is associated with its characteristic frequency band. The analyzed folding power (*AFP*) is a global characteristic of the hemispheric gyrification, very correlated to the sum of mean curvatures squared. This power is divided into 7 spectral bands with bandwidths of increasing (doubling) spatial frequency. The 4th, 5th, and 6th bands (B4, B5, and B6 respectively) gather more than 95% of total power and are associated with the variations of curvature related to the sulcal pattern (12). One of the interesting property of this spectrum is that variations in AFP may not divide equally between the 3 sulcal bands: for instance the increase in power that comes with increased brain size mainly add to the high frequency bands (to B6 more than B5 but not to B4) (12, 13).

Curvedness (*CVD*) and shape index (*SI*) were computed vertex-wise in each left cortical surface as follows, given the two principal curvatures *k1* and *k2*:

$$SI=\frac{2}{\pi }\tan^{-1}\frac{k_{2}+k_{1}}{k_{2}-k_{1}};CVD=\sqrt{\frac{{k_{2}}^{2}+{k_{1}}^{2}}{2}}$$

The meaning of what shape index and curvedness represent is proposed in Figure 2. Curvedness represents the local bending intensity (positive value), while shape index is a scale-invariant value canonically normalized between −1 and +1 that describes the local aspect of the surface. Sulci and gyri correspond to a value below −0.5 and above 0.5, respectively. As previously described (11), histograms of curvedness and shape index have characteristic shapes that can be summarized at the hemispheric scale by a few parameters (see Figure 3). Curvedness distribution was modeled by a gamma distribution from which the peak *c* was extracted as a global measure of the folding intensity. To obtain a dimensionless parameter as all the gyrification metrics we use otherwise, the normalized curvedness was defined as the product of *c* and *BV*^1/3^. The shape index distribution was modeled by a mixture of 2 beta distributions characterized by 2 parameters: the peaks *s1* and *s2* which represented the shape modes of sulci and gyri, respectively.

As segmentation errors were recurrent on the medial face of the cortical surface of patients due to periventricular WMH, we defined a lateral face by delineating a curve separating medial and lateral faces using Surfpaint Toolbox (19) in Anatomist (http://www.brainvisa.info). The morphometric descriptors of the cortex shape were computed on lateral faces only.

#### Effect of WMH-Induced Segmentation Errors

As we could not totally exclude that some WMH were not properly masked due to, for example, an imperfect registration between FLAIR and 3D T1-weighted images, we also tested the effect of adding simulated WMH in the white matter of controls as previously done (4). The idea was to differentiate the potential effect of WMH on the cortex shape in the disease from the effect of segmentation errors induced by WMH. To do this, patients were ordered according to their volume of WMH and one patient in two (*N* = 11) were selected. The 11 patients were sex- and age-matched to 11 controls. Then, 3D T1-weighted images of patients were non-linearly co-registered to 3D T1-weighted images of matched controls using FNIRT from FSL (www.fmrib.ox.ac.uk/fsl) (20). The matrix transform was applied to the mask of WMH so that the mask of WMH was registered in the controls space. Finally, mean gray level inside simulated WMH was set up to the mean gray level of the cortex for each subject (i.e., a very unfavorable setting for automatic segmentation algorithms) and gaussian noise was added to simulate a more realistic brain tissue. Thus, the simulation of WMH in the white matter of controls was supposed to produce much more segmentation errors than in the original conditions given that whole WMH of patients were transposed into controls brain, sometimes in regions very close to the cortex. T1-weighted images of controls including simulated WMH were *de novo* processed with BrainVISA to create new cortical surfaces prone to segmentation errors, from which curvedness and shape index were computed again.

### Statistical Analyses

Statistical analyses were conducted using the R software (http://www.r-project.org/). Between-group comparisons were performed using *t*-tests or X^2^ tests according to variable type and linear regression models including age, gender and brain volume as covariates (Table 1). Permutation tests were also used as a non-parametric alternative to compare morphometric outcomes. Finally, linear regression models were used in patients only to assess the links between morphometric parameters and WMH, independently of age and gender.

**Table 1:** Clinical characteristics of participants and results of global measurements, spectral, and morphometric analyses of the cortex shape.

	**CADASIL patients**	**Healthy controls**	***p*-value**
Number of subjects	21	28	
**Clinical characteristics**			
Gender, number of women (%)	13 (62%)	14 (50%)	0.59
Age, mean ± sd, range	54.5 ± 11.7, 32.1–74.5	53.8 ± 11.2, 30.1–71.4	0.83^a^
Level of education, mean ± sd (years)	10.7 ± 3.0	13.5 ± 3.5	**0.003^a^**
MMSE, mean, median, range	28.3, 29, 24–30	29.0, 29, 26–30	0.16^a^
**Global measurements (mean** **±** **sd)**			
Normalized brain volume	0.76 ± 0.03	0.76 ± 0.03	0.98^a^
L brain (GM + WM) volume, in cm^3^	486.6 ± 49.8	506.6 ± 45.4	0.16^a^
Global gyration index (*GGI*)	1.28 ± 0.08	1.29 ± 0.04	0.66^a^/0.88^b^
Spherical gyration index (*SGI*)	2.87 ± 0.23	2.89 ± 0.11	0.67^a^/0.98^b^
Volume of WMH, mean ± sd, range, in cm^3^	75.2 ± 55.6, 7.2–251.5	No significant lesions	–
Volume of lacunes^*^, mean ± sd, range, in mm^3^	467 ± 451, 14–1,356	No lacunes	–
**Spectral analysis (mean** **±** **sd)**			
Analyzed folding power *AFP*	303.0 ± 22.1	294.2 ± 15.0	0.13^a^/0.19^b^/**0.0005**^c^
B4 power	38.1 ± 2.2	36.8 ± 2.7	0.09^a^/0.14^b^/0.08^c^
B5 power	104.1 ± 7.1	101.7 ± 4.8	0.18^a^/0.15^b^/**0.01^c^**
B6 power	146.5 ± 15.1	145.0 ± 11.4	0.69^a^/0.79^b^/**0.04^c^**
**Morphometric analysis (mean** **±** **sd)**			
Normalized curvedness *c*	11.6 ± 0.7	11.1 ± 0.4	**0.003**^a^/ ** <0.0001**^b^/ <**0.0001**^c^
Sulcal shape index *s1*	−0.707 ± 0.008	−0.696 ± 0.007	**<0.0001**^a^/ ** <0.0001**^b^/ <**0.0001**^c^
Gyral shape index *s2*	0.550 ± 0.016	0.565 ± 0.010	**0.0006**^a^/ ** <0.0005**^b^/ **0.0003**^c^

a *t- test*;

b *asymptotic two-sample Fisher-Pitman permutation test*;

c *linear model with adjustment for age, sex and brain volume*;

*MMSE: Mini Mental State Examination; GM: gray matter; WM: white matter; WMH: White matter hyperintensities*;

* *computed in 12/21 (57%) having lacunes. Bold values means significant between-group differences (p < 0.05)*.

To test the effect of segmentation errors on morphometric measurements, we used a Wilcoxon signed-rank test as samples were small (*N* = 11) and paired between one control subject and one control subject with simulated WMH (Table 2).

**Table 2:** Mean observed effects of the disease and of WMH-induced segmentation errors.

**Morphometric parameter**	**MRD Cadasil – Controls**	***p*-value^a^**	**MRD Controls_**WMH**_ – Controls**	***p*-value^b^**
Normalized curvedness *c*	4.9%	0.003	0.9%	0.01
Sulcal shape index *s1*	−1.5%	<0.0001	−0.5%	0.03
Gyral shape index *s2*	−2.7%	0.0006	−1.2%	0.07

*MRD Cadasil—Controls: mean relative difference of morphometric parameters in Cadasil (N = 21) vs. Controls subjects (N = 28)*.

*MRD Controls_WMH_–Controls: mean relative difference of morphometric parameters in Controls with simulated WMH and segmentation errors (N = 11) vs. Controls subjects (N = 11)*.

a *t-test*;

b *Wilcoxon signed-rank test*.

## Results

### Effect of the Disease on the Cortex Shape

Clinical characteristics of the two groups and results of global measurements, spectral and morphometric analyses of the cortex shape are summarized in Table 1. Age and gender did not differ between the 2 groups conversely to educational level. Patients had similar brain volume, normalized brain volume and gyrification indices than healthy individuals, i.e., there was no significant atrophy of the brain parenchyma nor modification in gyrification intensity of the brain cortex (amount of buried cortical surface).

By contrast, the spectral and curvature-based morphometric analyses revealed significant differences between groups using either simple *t*-tests or linear models to adjust for potential confounders. Patients showed a higher total folding power (*AFP*) than controls. This increase in power divided up between the three sulcal bands, yet the difference between patient and control was significant only for B5 and B6. This indicates that the cortical folding of CADASIL patients required more high frequency components than the cortex of controls. Consistently, we found an increased curvedness *c* in patients, reflecting a more bended, sharper folding. The shape index *SI* brought complementary information: the shape of both sulci and gyri were different as *s1* and *s2* were smaller in patients compared to controls. Schematically, sulci were narrower while gyri were widened in patients (Figure 2). In terms of estimate accuracy, fitting errors for the gamma distribution of *c* and the beta distributions of *s1* and *s2* were <2 and 0.7% for both groups.

### Relationships Between Cortex Shape and the Volume of WMH

To determine whether potential differences in cortex shape were actually related to disease related mechanisms, we tested via a linear model the association between each cortex morphometric descriptor (*AFP, c, s1, s2*) and the volume of WMH, independently of age, gender, and other imaging parameters known to alter the cortex in CADASIL, namely the volume of lacune and normalized brain volume. There were no significant associations between *AFP, c* or *s1* and the volume of WMH. Gyral shape index descriptor *s2* was found to decrease with increasing volume of WMH (estimate = −1.79, std. error = 0.69, *p* = 0.02).

### Influence of Segmentation Errors on the Results

**Figure 4:**
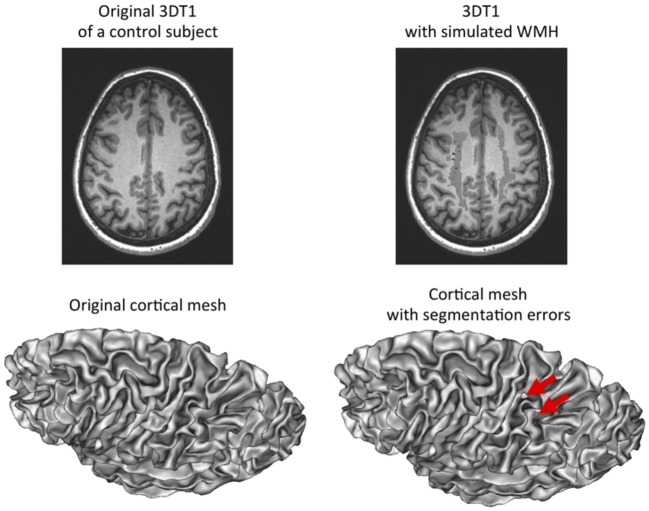
Simulation of white matter hypointensities in control subjects to induce segmentation errors. Left: original 3D T1-weighted image of a control subject with corresponding cortical surface. Right: same 3D T1-weighted image after addition of white matter hypointensites taken from an age-matched CADASIL patient after non-linear registration. The corresponding cortical surface presents with holes in the mesh as a result of segmentation errors due to the presence of white matter hypointensities close to the cortex and with similar gray-level values.

We have simulated WMH in T1-weighted images of controls to disentangle the effect of the disease from the effect of WMH-induced segmentation errors. As it can be observed in Figure 4, simulated WMH induce segmentation errors visible as holes and interruptions in the 3D mesh representative of the cortical surface. These errors were visually obvious which was expected since this simulation was supposed to provide an upper bound of the effects of segmentation errors in our data.

At the group level (11 controls vs. the same 11 controls with simulated WMH), we found a significant but small effect on *c* and *s1* (Table 2). In average, when adding WMH from different lesion loads sampled from the patient's group, *c* was increased by 0.9% while *s1* was decreased by 0.5%. By comparison, the difference between patient and control groups is 5 or 3 times greater for *c* and *s1* respectively, while expected to be less affected by WMH induced segmentation errors than by simulated ones. There was no significant effect of simulated WMH on *s2* (Table 2), the parameter that has been found related to the volume of WMH in patients.

## Discussion

In the present study, we found that the cortex of CADASIL patients was characterized by a surface that is more sharp and more bended through both the spectral and geometrical analyses, with a difference in global shape of folds toward somehow “tightened” sulci and “expanded” gyri. Also, the level of alteration of the gyral shape descriptor was significantly associated with the extent of WMH that reflects the amount of white matter tissue changes in CADASIL. Altogether, these results support the hypothesis of a gyral white matter swelling.

Our main hypothesis to explain these results is mechanical: the white matter swelling would be “pushing” the cortex so that its shape is slightly modified. As represented in Figure 1, the cortex expansion might be limited by the meningeal envelope, favoring the shape alteration in the tangential direction. One can figure out this diffuse and global mechanism by imagining an increase in the white matter volume inside a gyrus beneath the cortex. As a consequence of increased pressure, the gyrus expands (decreased positive *s2*) and adjacent sulci tighten (decreased negative *s1*) due to lateral compression. This hypothesis could be modeled by a combination of the recent mechanical model by Tallinen et al. (5) and the model by Nie et al. (6) that is able to reproduce the interaction between gray matter and meninges. As for the spectral parameters, the global increase in *AFP* may be due to sharper sulci bank and gyri edges that generate more high frequency component in the spectral decomposition, resulting in more *B5* and *B6* power that are not explained in our data by an increased in cortical surface buried in *B5* and *B6*-associated pieces of sulci (data not shown). Indeed in Fourier-like decomposition, higher frequencies not only contribute to periodic patterning but also to sharpness rendering. Another explanation is that the small increase in global folding power really divides-up into the three sulcal bands because it is a global effect not related to an increase in gyrification complexity nor an extension of the folding pattern.

By simulating WMH in cerebral images of controls, we have shown that segmentation errors due to uncorrected WMH near the cortex could slightly change the values of some descriptors of the cortex shape we used. However, these changes were non-significant (*s2*) or much smaller (*c, s1*) than the differences observed between groups. These results were convincing given that the simulated WMH produce much more obvious errors than our original data. Therefore, we could conclude that the observed differences were mainly driven by the disease while we could not exclude a small effect of segmentation errors on *c* and *s1*.

Our study owns some limitations and questions still open to interpretation. First, direct involvement of the cortex microstructure could also be involved in the alteration of the cortex shape aside with gyri white matter swelling. Yet, no significant differences of cortical thickness have been reported in this cohort of paucisymptomatic CADASIL patients compared to age- and sex-matched controls (21). Similarly, no visible cortical infarcts have been detected. Second, there was a significant statistical association between *s2* and the volume of WMH that we assumed to reflect the effect of white matter swelling on cortex shape, but we did not truly measure the volume of white matter. In fact, we considered global volumetric methods inappropriate to accurately detect a change at the group level. More, even after correction there may be still uncorrected WMH in T1-weighted images due to imperfect registration from FLAIR to T1-weighted images that may corrupt the white matter volume measurement. Ultimately, we found a similar normalized brain volume between groups, which is in favor of a swelling mechanism that would balance the expected cerebral atrophy observed in sporadic small vessel diseases (22). Longitudinal MRI follow-up and evaluation of the metrics presently used to characterize the cortex shape may be a future step to better understand the course of those alterations. Additional sources of data may also be helpful to decipher these mechanisms. For example, combining cortex shape analysis and structural connectome analysis derived by reconstructing fiber pathways with diffusion MRI may reveal some relationships between connected areas. Also, region-based analysis with dedicated methods could be useful to characterize local alterations in cortex shape and determine whether some regions are more affected than others (23).

In conclusion, the shape of the cortex of CADASIL patients is altered compared to age- and sex-matched controls at an early stage of the disease. This cortical alteration is partly associated to the swelling of the underlying white matter. More generally, the study of the cortex shape might be another way to evaluate subcortical swelling or atrophy in some pathological conditions strongly affecting subcortical tissues. New computational tools assessing cortical shape may help revealing subtle preclinical structural changes where global measurements might fail. Future studies are needed to assess the association between metrics of cortex shape and cognitive scores and clinical outcomes.

## Ethics Statement

This study was carried out in accordance with the recommendations of Good Clinical Practice and applicable local regulations with written informed consent from all subjects. All subjects gave written informed consent in accordance with the Declaration of Helsinki. The protocol was approved by a local ethics committee (Ile de France VII, No. CPP09-012).

## Author Contributions

FD and EJ: study concept and design. EJ: acquisition of data. FD, DG, JL, CF, and EJ: analysis and interpretation of the data. FD and EJ: drafting of the manuscript. HC and EJ: study supervision. All authors: critical revision of the manuscript.

### Conflict of Interest Statement

The authors declare that the research was conducted in the absence of any commercial or financial relationships that could be construed as a potential conflict of interest.
